# Typical Pediatric Brain Tumors Occurring in Adults—Differences in Management and Outcome

**DOI:** 10.3390/biomedicines9040356

**Published:** 2021-03-30

**Authors:** Ladina Greuter, Raphael Guzman, Jehuda Soleman

**Affiliations:** 1 Department of Neurosurgery, University Hospital of Basel, 4031 Basel, Switzerland; raphael.guzman@usb.ch (R.G.); jehuda.soleman@usb.ch (J.S.); 2 Division of Pediatric Neurosurgery, University Children’s Hospital of Basel, 4031 Basel, Switzerland; 3 Faculty of Medicine, University of Basel, 4056 Basel, Switzerland

**Keywords:** adult brain tumors, pediatric brain tumors, medulloblastoma, pilocytic astrocytoma, craniopharyngioma

## Abstract

Adult brain tumors mostly distinguish themselves from their pediatric counterparts. However, some typical pediatric brain tumors also occur in adults. The aim of this review is to describe the differences between classification, treatment, and outcome of medulloblastoma, pilocytic astrocytoma, and craniopharyngioma in adults and children. Medulloblastoma is a WHO IV posterior fossa tumor, divided into four different molecular subgroups, namely sonic hedgehog (SHH), wingless (WNT), Group 3, and Group 4. They show a different age-specific distribution, creating specific outcome patterns, with a 5-year overall survival of 25–83% in adults and 50–90% in children. Pilocytic astrocytoma, a WHO I tumor, mostly found in the supratentorial brain in adults, occurs in the cerebellum in children. Complete resection improves prognosis, and 5-year overall survival is around 85% in adults and >90% in children. Craniopharyngioma typically occurs in the sellar compartment leading to endocrine or visual field deficits by invasion of the surrounding structures. Treatment aims for a gross total resection in adults, while in children, preservation of the hypothalamus is of paramount importance to ensure endocrine development during puberty. Five-year overall survival is approximately 90%. Most treatment regimens for these tumors stem from pediatric trials and are translated to adults. Treatment is warranted in an interdisciplinary setting specialized in pediatric and adult brain tumors.

## 1. Introduction

In children, brain tumors are the most frequent solid cancer observed, with an incidence reported of 5.7 per 100,000 children, while in adults, the incidence is much higher with 29.9 per 100,000 persons [1]. Pediatric and adult brain tumors do not only differ in their incidence but also in histology, molecular pathology, location, and outcome [1]. However, some typical pediatric brain tumors, at times, occur also in adults. Of these, germinomas, non-germinomatous germ cell tumors or pineal region tumors show comparable behavior or occur equally often in children or adults [2,3,4,5,6,7]. On the contrary, medulloblastoma, pilocytic astrocytoma, and craniopharyngioma differ essentially in their incidence, location, histology and molecular pathology, treatment strategies, or outcomes between the two age groups. It is to be assumed that the knowledge of how to deal with these pediatric brain tumors might be limited within the adult word since their incidence is lower in the adult population. The aim of this review is to give an overview of these three classical pediatric brain tumors and emphasize on their incidence, behavior, classifications, treatment strategies, and outcome in the adult population, while comparing to their pediatric counterparts.

## 2. Medulloblastoma

### 2.1. Incidence and Classification

Medulloblastoma (MB) is the most common malignant brain tumor in children, with an incidence of 5–6 cases per million children. However, in adults, medulloblastoma is a very rare tumor, accounting for only 0.6 cases per million persons. MB can be associated with cancer predisposition syndromes, such as Gorlin, Turcot, or Li Fraumeni syndrome [8]. The biology of MB with different molecular and genetic subgroups varies across the different age groups, and creates subgroup- and age-specific patterns with different outcome [2,7,9]. Historically, MB were divided according to the Chang classification for local tumor invasion (T stage) and metastasis (M-stage) [10]. In adults, prognostic association of T-stage and outcome was found, while in children, no such evidence exists [2,11,12]. 

According to the WHO brain tumor classification, MB are by definition WHO grade IV tumors regardless of their subtype [13]. Histologically medulloblastomas are divided into three main subtypes: classic, nodular/desmoplastic, or large cell/anaplastic [13]. Additionally, extensive nodular histology, exclusively existing in infants, and not otherwise specified (NOS) MB were added to the WHO classification [13,14,15]. In the previous WHO guidelines large cell and anaplastic histology were classified separately, however, large cell medulloblastomas mostly also show anaplastic features, which is why these types were grouped together [13,15,16]. Nodular/desmoplastic histology was described to occur more frequently in adults and is associated with a lower risk classification than classic or anaplastic types in both age groups [17]. Additionally, with the development of genetic and molecular analysis, new subgroups have been defined in the last decade. According to the consensus conference in 2016, medulloblastomas are divided into three main subgroups according to their genetic and molecular changes: wingless-type (WNT), sonic hedgehog (SHH), and non-WNT/non-SHH with Group 3, and Group 4 [13,18]. Recently, new sub-classifications for Group 3 and Group 4 medulloblastomas were added based on DNA methylation profiling [19]. Several additional prognostic factors based on gene expression were identified, such as *MYC* in Group 3 MB and *MYCN* amplification in Group 4 MB (poor prognosis), and *TP53* mutation (poor prognosis) in adult SHH-MB [2,7,20].

SHH-MB *TP53* wild-type is the most common subgroup found in adults [20,21]. It shows a male predominance (2:1), and accounts for around 60% of adult MBs and is significantly associated with nodular/desmoplastic histology [11,21,22,23,24]. WNT-MB occurs in approximately 10–15% of adults and displays classic histology in 95%. WNT-MB shows the best overall survival, however, in adults a more aggressive behavior is observed than in children [21,23,25]. The remaining 25% of adult MB are represented by non-SHH non-WNT MB, mostly belonging to Group 4-MB. In adults, a third of Group 4-MB show anaplastic histology and metastatic disease, both factors which are associated with a poor prognosis [23,25,26]. Group 3-MB are predominantly found in infants and were only later on described in adults, in which they are a rare occurrence [20,22,23,25]. An overview of the different subgroups and their differences in adults and children is shown in Table 1.

### 2.2. Clinical Presentation and Diagnostic Tools

**Figure 1:**
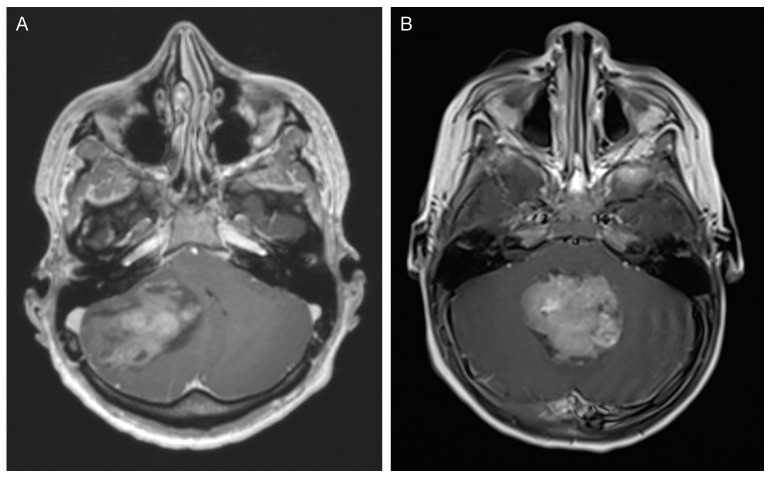
(**A**) 27-year-old male, presenting with headache and ataxia. Axial MRI shows a lateral cerebellar mass with concomitant hydrocephalus. He recovered well after surgical tumor resection. Histopathological analysis diagnosed an SHH-MB, classic histology, TP53 wild-type. He received CSI and chemotherapy according to the Packer regiment [31]. (**B**) 3-year-old female, presenting with vomiting and unsteady gait. Axial MRI shows a lesion in the 4th ventricle with obstruction and hydrocephalus. Preoperative CSF diversion was installed and after complete resection, the patient required a ventriculo-peritoneal shunt and recovered well. Histopathological analysis showed a Group 3 MB, *MYC* amplification negative, anaplastic histology. Further work-up showed a spinal lesion suggestive of metastasis (not shown). She received chemotherapy according to the HIT-study regiment [32].

In adults, MB are mostly localized in the cerebellar hemisphere (SHH-MB) resulting in gait ataxia (68%) and/or vestibular syndromes (41%), while in children, MB mostly occurs in the midline and causes hydrocephalus due to an obstruction of the 4th ventricle (Figure 1). 

Due to the mass effect and elevated intracranial pressure in the posterior fossa, 80–90% of adults and children suffer from headaches and vomiting [33,34,35]. Most symptoms have been present for a longer period of time (approximately 2 months) at the time of diagnosis, but especially in children, who often present with unspecific symptoms and psychomotor regression at first, the delay between symptom onset and diagnosis can reach up to several months [33,34,36]. 

Magnetic resonance imaging (MRI) is the method of choice for diagnosis. MB presents as iso- to hypointense mass on T1 and hypo- to hyperintense on T2 images. It enhances with contrast and shows an increased signal on diffusion-weighted imaging series (DWI) [2,37]. Adult MB shows a more irregular contrast enhancement compared to pediatric MB, which allows for a correlation between imaging and molecular subtype [37]. Similar to the pediatric population, also in adults, a complete neural axis, including spinal axis, MRI imaging with contrast is recommended [2,38]. 

### 2.3. Management and Outcome

In both children and adults, surgery is the primary therapy. Depending on the individual case a temporary CSF diversion (extraventricular drain [EVD] or endoscopic third ventriculostomy [ETV]) needs to be performed primarily [2]. Permanent CSF diversion with a ventriculo-peritoneal shunt is indicated in 20% of all medulloblastoma patients, but is significantly lower in patients suffering from WNT-MB [39]. In general, young age (<2 years) is a risk for developing shunt dependency after surgery of a posterior fossa lesion and children (20–40%) require permanent shunting more often than adults (7–21%) [40,41,42]. The primary aim of surgery is gross total resection, however, since MB can invade the floor of the 4th ventricle, a maximal safe resection is key for a good outcome. Since MB in adults is more often localized to the cerebellar hemisphere (especially SHH-MB) a gross total resection is achieved more often. A remaining tumor volume of <1.5 cm^3^ should be aimed for if feasible, as it is of positive prognostic value in children [43]. The surgical approach chosen is similar in adults and children and depends on the individual tumor location. In children, however, special care should be taken to spare the vermis. Children tend to develop posterior fossa syndrome, a combination of mutism, cranial nerve deficits, and emotional lability, after manipulation of the vermis (referred to as “posterior fossa syndrome”), while this phenomenon is less often observed in adults [44,45]. To avoid extensive traction of the cerebellum, a telovelar approach is recommended for midline tumors, which can either be done in a prone, park-bench, or sitting position, depending on the surgeon’s preference [2]. 

Postoperative outcomes mostly depend on the molecular subgroup of MB, as mentioned above. However, regardless of the subgroup, to this date, craniospinal irradiation (CSI) with a boost to the posterior fossa is recommended for all patients above the age of 3 years. Deescalating therapies depending on molecular subtypes, especially for more favorable subtypes like WNT- and SHH-MB, are being studied. Most studies investigating CSI in medulloblastoma, were carried out in the pediatric population, while their results are extrapolated to the adult population. CSI was shown to significantly increase progression-free survival (PFS) and overall survival (OS) in MB and should be commenced within 6 weeks after surgery [2,9,12,46]. 

Additional to CSI, chemotherapy is recommended for MB irrespective of the tumor subgroup or the patient’s age [27]. Young children receive a combination of cyclophosphamide, vincristine, carboplatin, etoposide and intrathecal methotrexate, according to the HIT 2000 regiment [28]. Chemotherapy regimens for adults are adapted from pediatric trials, and are based on a regiment (Packer regiment) with vincristine, followed by lomustine (CCNU), and cisplatin [28,31,47]. Adolescents and adults, however, show a lower tolerance and higher toxicity to chemotherapy compared to children, and individual adaptions to the standard protocol might be required [2,29]. In adults, a protocol by Franceshi et al. using cisplatin or carboplatin plus etoposide, showed an improvement of the PFS at 15 years in patients treated with radiotherapy and chemotherapy (PFS 82.3% ± 8.0%) compared to patients treated with radiotherapy alone (PFS 38.5% ± 13.0%, *p* = 0.05) [27]. Adult MB shows a tendency towards late recurrences (>5 years after initial diagnosis), hence, a long-term follow-up is recommended [11,33]. In the treatment arm of the study by Franceshi et al., OS for adult MB at 10 years is reported at 89% [11,27]. In general, adults have a worse OS compared to children with MB, but the molecular subtype is of paramount importance in assessing the individual prognosis in children and adults [18,27], (Table 1 and Table 2). 

Novel targeted therapies on a molecular level, might potentially change the treatment regimens and prognosis of medulloblastoma in children and adults [48,49]. SHH-MB can be targeted with Smoothened (SMO) inhibitors, such as sonidegib and vismodegib [30]. So far only phase I and II trials in recurrent MB are available [30]. A study with sonidegib in children and adults showed a tumor response in SHH-activated medulloblastomas, however, in children the drug was discontinued early due to its inhibitory effect on skeletal growth plates [50]. Similar results were described for vismodegib [51]. However, it has to be considered that SHH-MB are rare in children, and these drugs might have a limited use in this age group, while they could hold promising results in adults [30].

## 3. Pilocytic Astrocytoma

### 3.1. Incidence and Classification 

Pilocytic astrocytoma (PA, also known as juvenile pilocytic astrocytoma) is one of the most common brain tumor found in children, comprising 15% of all pediatric brain tumors [52]. In adults however, PA is less frequent with an incidence of 0.1 per 100,000 persons compared to 0.8 per 100,000 children [5,6]. Children suffering from neurofibromatosis type 1 (NF-1) and tuberous sclerosis (TS), two cancer-predisposition syndromes, show a higher rate of low-grade gliomas. Children with TS develop subependymal giant cell astrocytoma, a specific subtype of astrocytoma, while children with NF-1 show a predilection for optic gliomas, a subtype of PA [6,52], while in adulthood patients with NF-1 tend to develop high-grade gliomas [53,54]. In general, PA can occur anywhere in the central nervous system, but the classical childhood PA occurs in the cerebellum, while in adults, it is found in the supratentorial compartment. Highly eloquent localization in the brainstem is observed in around 10–20% in children and in around 5% in adults, while spinal manifestation occurs in approximately 2–5% in both age groups [55,56,57]. PA is by definition a WHO Grade I tumor [13]. It differs from other low-grade gliomas (LGG), as it is not a precursor of diffuse gliomas, which occur in both age groups and tend to undergo malignant transformation, especially in adults [13,58,59]. Histologically, PA shows areas of compact astrocytes and Rosenthal fibers and areas of loosely textured cells. Proliferation indices like Ki-67 are around 4% and anaplasia is rarely observed in PA but occurs more frequently in NF-1 patients or older patients [60,61,62]. Histopathological analyses have shown that an activation of the PI3K/AKT pathway might increase the aggressiveness of PA, leading to higher recurrence rates and lower overall survival [61,62]. *MGMT* promoter methylation was also discovered in over half of anaplastic PA but not in PA in general, however, no association of *MGMT* promoter methylation with outcome was shown so far [62]. Molecular analysis detect a fusion or mutation of the *BRAF* gene in up to 70% of childhood PA, which might activate oncogenic pathways, and could have prognostic implications [63,64,65]. However, *BRAF* fusion was only found in 20% in adult PA, indicating a decrease of *BRAF* fusion with increasing age [66]. 

### 3.2. Clinical Presentation 

Children and adults mostly present with headache, nausea, and vomiting, due to elevated intracranial pressure. Additional symptoms like motor deficits or ataxia are dependent on the individual tumor location. Despite hydrocephalus present at the time of diagnosis, only a few patients need preoperative CSF diversion. Especially in children, symptoms were retrospectively present for several months already, until the definitive diagnosis was made [36,67].

Initial diagnosis is mostly made by MRI, which in the posterior fossa usually presents as a cyst with a mural tumor nodule. The solid nodule is T1-hypointense and T2-hyperintense compared to the brain tissue, and the cyst wall mostly enhances with contrast [68]. In the spinal cord, PA also presents as a cystic and nodular tumor, which enhances with contrast and mostly shows an eccentric growth pattern [69]. 

### 3.3. Management and Outcome

The primary therapy of PA is gross total resection (GTR) of the tumor, which leads to excellent PFS and OS. However, for deeply or eloquently seated lesions (brain stem, optic tract), usually, only a partial resection is feasible, and in most cases the cyst wall is mostly benign and can be left intact [70,71]. These patients were shown to have a higher risk for mortality compared to patients with cerebral or cerebellar tumor location [6,56,72]. So far, GTR is the only treatment in adults, which showed a benefit in survival [6,73]. Radiotherapy in adult and childhood PA is controversially discussed [6,56]. No interventional trials for radiotherapy in PA exist. In current practice, most patients receiving radiotherapy have either a deep-seated lesion, recurrence, or discordant histopathology, which influences the outcome of observational studies towards a shorter PFS in patients receiving radiotherapy [6,56,74]. Radiotherapy is avoided in very young children or children suffering from NF-1, due to their increased risk of irradiation-induced cell damage and the potential of malignant transformation of the tumor [52,59]. Chemotherapy is mostly administered at recurrence. In a retrospective cohort study, only 13% of adults and children received radio- or chemotherapy postoperatively, while the administration of chemotherapy increases for patients with optic or brainstem PA or only partial tumor removal [75]. Chemotherapy in adults consists either of temozolomide or a regimen of carboplatin, etoposide, and vincristine [56], while in children, cisplatin, vincristine, or vinblastine are administered [76]. Recently, with the development of molecular analysis and pathway recognition, novel targeted therapies with MEK-inhibitors are being tested, showing promising results. However, as *BRAF* mutations are rare in adults, these novel targeted therapies remain reserved mainly for childhood PA [77,78]. 

In general, adults show a more aggressive behavior of PA than children, with recurrence rates of over 30%. Five-year overall survival for adult PA is estimated at 83–87%, while for pediatric PA a 5-year overall survival of 95% can be reached [6,55,56,57]. An overview of the characteristics of adult and childhood PA is shown in Table 3. 

## 4. Craniopharyngioma

### 4.1. Incidence and Classification 

Craniopharyngiomas show a bimodal age distribution with a peak between 5–14 years and a second peak in adults between 50–70 years. The overall incidence is 0.13–0.18 per 100,000 persons in adults and children, while it seems that the incidence is similar in both children and adults [79,80]. Craniopharyngiomas constitute around 4–9% of pediatric brain tumors and 2–5% of adult intracranial tumors [81,82]. According to the WHO classification of tumors, craniopharyngiomas are regarded as histopathologically benign lesions (WHO I) [13]. The two major histopathological subtypes of craniopharyngioma are adamantinomatous (ACP) and papillary (PCP) [83]. The first can occur in any age group, but is predominantly found in children, while the latter is only found in adults [82,84,85]. ACP mostly presents with macroscopic cysts, filled with cholesterol-containing fluid, and calcifications, while PCP present as solid tumors. Both types differ concerning their oncogenic genetic alterations, with changes in the Wnt/B-catenin pathway due to a *CTNNB1* mutation for ACP and a *BRAF* mutation for PCP [85,86,87]. No differences in methylation profiles between adult and pediatric ACP were described [88].

### 4.2. Clinical Presentation

Craniopharyngiomas cause symptoms due to their intrasellar location and suprasellar growth, resulting in compression or invasion of the surrounding structures (optic nerve/chiasm, pituitary, hypothalamus, 3rd ventricle). The grade of hypothalamic involvement can be classified on preoperative MRI according to Puget et al. in Grade (1) no hypothalamic involvement, Grade (2) hypothalamic displacement, and Grade (3) hypothalamic invasion [89]. Despite similar tumor location, main clinical symptoms differ between children and adults. In adults, the main symptom at diagnosis are visual field deficits, while these are detected later in children [90]. Bitemporal hemianopia due to chiasmal compression is the most common visual field deficit and is found in around 60% of all patients with CP. Typical endocrinological deficits due to compression of the pituitary stalk occur in over 60% of children but only in around 30% of adults [90]. Endocrine deficits in children often manifest with a short stature or delayed puberty [82]. Froehlich’s Syndrome is a combination of hypogonadism and obesity due to a hypothalamic pituitary pathway failure observed only in childhood craniopharyngioma [87,89]. Adults mostly present with more subtle hormonal deficits, which might be only found in laboratory examinations [82,91,92]. 

### 4.3. Treatment Strategies and Outcome 

**Figure 2:**
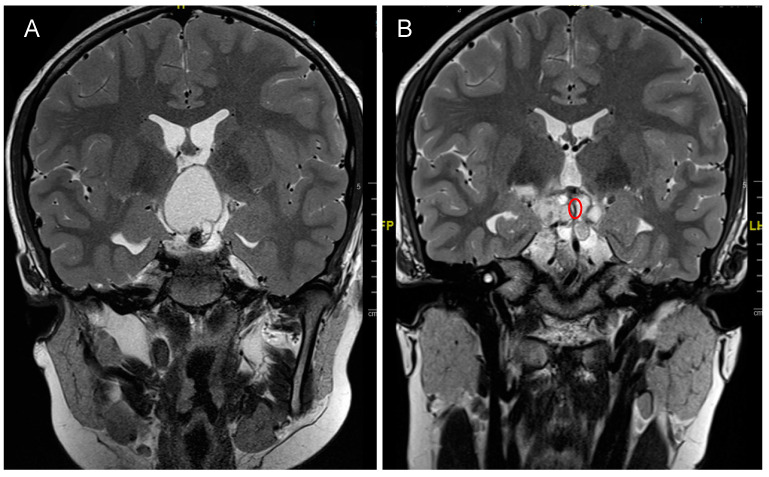
12-year-old male, presenting with growth delay and obesity due to endocrine deficits caused by an adamantinomatous craniopharyngioma, Puget 2. (**A**) Preoperative coronal image showing a space-occupying cyst of the CP causing hydrocephalus. (**B**) Postoperative coronal image showing drained cyst and the tip of the inserted drain (red circle). The patient was then treated with proton beam therapy as an adjuvant treatment to surgery.

In both age groups, the main treatment consists of surgical resection. The planned surgical resection depends on the anatomical extension of the tumor, but in adults generally the aim is for a GTR or near total resection (NTR, >90% of tumor volume), while cyst drainage alone is rarely observed [90]. However, in children, the optimal treatment strategy is more controversially discussed [3,93]. It should mainly aim for a relief of symptoms ensuring local tumor control, while preserving a high quality of life, and not ultimately aim for a complete resection risking endocrine deficits caused by hypothalamic injury [89]. Postoperative hypothalamic injury can be classified in the same fashion as preoperative invasion [89]. Especially in children with a Puget grade 2 hypothalamic invasion, the goal of surgery should be tumor reduction or cyst drainage and not GTR [89]. Children with hypothalamic impairment, have a very high rate of hyperphagia, obesity, neurocognitive deficits, and lower quality of life, and therefore one of the main goals during surgery in children is preservation of the hypothalamus [89,93,94,95]. The preservation of the hypothalamus and also pituitary stalk during surgery reduces the rate of postoperative diabetes insipidus, but could cause a higher risk for tumor recurrence in both children and adults [82,96,97,98]. For these reasons, more and more pediatric neurosurgeons consider a tumor cyst fenestration and aspiration with tumor biopsy (endoscopically or stereotactically) as the primary treatment since it improves the symptoms and causes significantly lower overall morbidity compared to GTR [3,99,100]. After cyst fenestration, a drain can be left in-situ and connected to an Ommaya reservoir, which allows for repeated cyst aspirations (Figure 2). 

**Figure 3:**
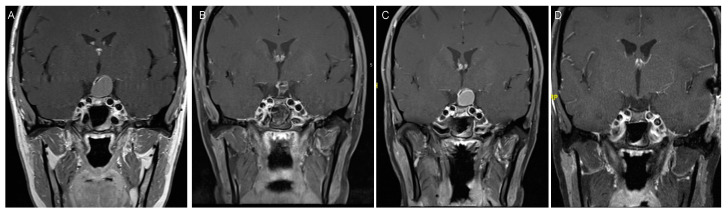
27-year-old female patient, presenting with amenorrhea and disturbed peripheral color vision. (**A**) MRI for further analysis showed a cystic tumor with compression of the pituitary gland. (**B**) She first underwent transsphenoidal cyst fenestration and partial resection, which she recovered from well with full recovery from her visual deficits. Histopathology diagnosed an adamantinomatous CP. (**C**) Within 6 months, a progression of the cyst was observed, and she again developed visual field deficits and disturbed color vision. (**D**) She then underwent pterional craniotomy for complete tumor removal and additionally received proton beam therapy as adjuvant therapy. Her visual field deficits improved over time, while her endocrine deficits persisted, requiring hormonal substitution. Nowadays, the pediatric approach with neuroendoscopic cyst fenestration followed by proton beam therapy could have been applied for this case, while craniotomy is the more traditional approach.

If the procedure is done endoscopically, STR can be achieved as well, leading to a reduction of the tumor volume as preparation for the proton beam therapy, which is administered as an adjacent measurement to surgery [101]. Also, in adults, endocrine deficits are often observed with radical resections of invasive tumors affecting the hypothalamus and pituitary stalk, however, this does not result in any developmental impairments as observed in children, but rather in endocrine dysfunctions, which are then treated with hormonal replacement medication [85,90,93]. For surgery in adults, either an open transcranial or transsphenoidal approach can be chosen (Figure 3). 

However, some authors show in adults with cystic craniopharyngiomas similar surgical strategies as in children with cyst fenestration, tumor reduction, Ommaya reservoir, and radiation [102,103]. In CPs with a large lateral or cranial extension, transsphenoidal approaches might be limited due to the carotid arteries or suprachiasmatic location [104]. If a transsphenoidal approach is chosen in children, one has to consider, that the pneumatization of the sphenoid sinus only starts after the age of 3 years and is not fully developed until the age of 12–14 years, limiting these approaches in young children [105]. A literature review by Komotar et al. showed a significantly higher extent of resection with either microsurgical or endoscopic transsphenoidal approach compared to open transcranial surgery, promoting transsphenoidal approaches [106]. STR has a recurrence rate of up to 100%, however, even if GTR is assumed, recurrences can be observed in up to 20% of the cases [3,84,102,107]. In addition, GTR was not shown to correlate significantly with OS in adults. This promotes the strategy of a planned partial resection, especially in elderly patients, and patients with a known hypothalamic involvement, to reduce endocrine deficits postoperatively [90]. In summary, for the adult population the surgical strategy for non-cystic CP is transsphenoidal resection whenever possible, while GTR should be the goal of surgery. For cystic CP, traditionally, an open transcranial or transsphenoidal approach was the treatment of choice. To date, more and more authors adopt the surgical strategy used in the pediatric population of endoscopic cyst fenestration, tumor reduction and adjuvant proton beam therapy, since the morbidity of surgery is significantly lower. Further studies in adults are still needed to show that this treatment strategy concerning PFS, and OS is similar in adult as in the pediatric population.

Postoperative radiotherapy as fractionated radiotherapy, radiosurgery, or proton beam therapy is described for local tumor control in adults, however, is mostly restricted to smaller tumors or residual tumor after surgery. Local tumor control with fractionated radiotherapy is best achieved with doses around 54–55 Gy, because with increased doses an elevated risk for endocrine side effects is observed [108]. In children, proton beam therapy showed a lower rate of radiation to the surrounding structures, especially to the optic apparatus and hypothalamus, compared to conventional radiotherapy and achieves a good local tumor control [103,109,110]. Hence, it is often used in combination with tumor reduction surgery or cyst drainage to ensure local tumor control, without risking hypothalamic injury by extensive surgery [101]. No instillation of chemotherapy or similar substances (bleomycin, radioisotopes, interferon alpha) has been described in adults, and these experiences are solely based on pediatric populations with mixed results [93,99,108]. In PCP, therapy targeting the *BRAF* mutation with Dabrafenib or Vemurafenib showed a good radiographic response and tumor control in a few cases [111]. In ACP, targeted therapies with MEK inhibitors could have a certain therapeutic potential, however, reports are scarce [112,113]. Further trials with targeted therapies in CP are needed in the future. 

In general, CP has an excellent 5-year survival rate of 90% in both pediatric and adult population [84,90,102]. An overview of the characteristics of pediatric and adult CP is shown in Table 4. 

## 5. Conclusions

The same tumor entities, namely medulloblastoma, pilocytic astrocytoma and craniopharyngioma, show differences in their incidence, histopathological and molecular features, treatment, and outcome between children and adults. This requires individual management strategies for the different age groups. 

In MB the distribution of the different subtypes varies among the age groups and has implication on the individual prognosis. However, surgery remains the primary therapy in most cases, followed by radio- and chemotherapy. 

In adults, PA is mainly a supratentorial tumor, while in children it is a commonly found infratentorial. GTR is the primary therapy for PA in both age groups and correlates with a benefit in survival. Radio-or chemotherapy is only administered in around 10% of all cases and is reserved for recurrent tumors. 

CP with its intra- and suprasellar location, mostly causes symptoms due to compression and invasion of the surrounding structures. In adults the presenting symptoms are bitemporal hemianopia, while children mostly present with endocrine deficits. Surgery with the aim to reduce the mass effect and achieve a complete resection is the primary treatment, however, if hypothalamic invasion is presented an intended subtotal resection followed by proton beam radiation was shown to have fewer side effects. 

Currently, most treatment strategies for these tumors stem from pediatric trials and are translated to adults, due to a paucity of data in the adult cohort. Therefore, and due to the complexity and distinct features of these tumors, interdisciplinary management and discussion with teams specialized in pediatric neurosurgery, pediatric neuro-oncology and pediatric neuropathology, on how to diagnose, treat, and follow up these patients is warranted. 

## Figures and Tables

**Table 1:** Characteristics for the molecular subgroups of adult and pediatric medulloblastoma, based on data from the following references [2,7,8,9,11,13,18,21,22,23,24,25,27,28,29,30]. Abbreviations: CPA = cerebellopontine angle, SHH = sonic hedgehog, WNT = wingless, OS = overall survival.

	Adults				Children			
Subgroup	SHH	WNT	Group 3	Group 4	SHH	WNT	Group 3	Group 4
% of cases	60–65%	10–15%	5%	20%	20–25%	10–15%	20–25%	40%
Gender Ratio (m:f)	2:1	1:1	2:1	4:1	2:1	1:1	2:1	1:1
Location	Cerebellar hemisphere/ CPA	Cerebellar hemisphere/ CPA	Midline, 4th ventricle	Midline, 4th ventricle	Cerebellar hemisphere	Cerebellar hemisphere	Midline, 4th ventricle	Midline, 4th ventricle
Histology	Nodular-desmoplastic	Classic	Classic	Classic/ Anaplastic	Classic/ Nodular- desmoplastic/ Anaplastic	Classic	Classic/ Anaplastic	Classic/ Anaplastic
Metastasis (%)	<10, local	<10, local	10–15, distant	20, distant	10–15, Local	<10, Local	40, Distant	35, Distant
Molecular/Genetic alterations	TP53 (poor prognosis)	TP53	MYC N *	MYC *	TP53 (poor prognosis) MYC N	-	MYC N	MYC
Prognosis	Intermediate, Poor with TP53	Good	Poor	Intermediate	Intermediate, Poor with TP53, Infants better	Excellent	Poor	Intermediate
5-year OS (%)	81% TP53: 41%	82%	25%	39%	75–90%, TP53: 40–50%	>90%	55%, MYC N: <50%	75–90%

* rare in adults.

**Table 2:** Overall characteristics for adult and pediatric medulloblastoma based on the following references [8,11,12,27,29,33,34,35,40,41,42,44,45] Abbreviations: CSI = craniospinal irradiation.

Characteristics	Adults	Children
**Incidence per 10^6^ persons**	0.6	5–6
**Location (most common)**	Cerebellar hemisphere	Midline, 4th ventricle
**Presenting Symptom**	60% gait ataxia, 40% vestibular syndrome, >80% hydrocephalus	>80% vomiting, hydrocephalus
**Associated Syndromes**	-	Li-Fraumeni, Gorlin, Turcot
**Molecular alterations**	Depending on subtype	Depending on subtype
**Metastasis**	Depending on subtype	Depending on subtype
**Primary Treatment**	Surgery	Surgery
**Additional Therapy**	Chemotherapy (Packer regiment), CSI	Chemotherapy (HIT 2000 regiment), CSI (>3 years)
**Posterior Fossa Syndrome postoperative (%)**	16	8–39
**Shunt Dependency (%)**	7–21	20–40
**Prognostic Factors**	Depending on subtype	Depending on subtype
**5-year OS (%)**	25–82%	50–90%

**Table 3:** Characteristics for adult and pediatric pilocytic astrocytoma, based on data from the following references [5,6,13,54,55,56,57,58,64,65,66,75]. Abbreviations: OS = overall survival, NF = neurofibromatosis, GTR = gross total resection, STR = subtotal resection, MEK inhibitor = mitogen-activated protein kinase.

Characteristics	Adults	Children
**Location (most common)**	supratentorial (35–45%), cerebellar (35–40%), brain stem, optic pathway (5–10%), spinal (2–5%)	cerebellar (70%), brain stem, optic pathway (10–20%), spinal (2–5%)
**Associated Syndromes**	-	NF-1, Tuberous Sclerosis Complex
**Molecular alterations**	BRAF: 20%	BRAF: 70%
**Primary Treatment**	Surgery	Surgery
**Additional Therapy**	Chemotherapy (temozolomide, carboplatin, etoposide), Radiation for deep-seated lesions, recurrence	Chemotherapy (cisplatin, vincristine, or vinblastine), Radiation (>3 years) for deep-seated lesions or recurrence, MEK inhibitor for BRAF mutation
**Prognostic Factors**	GTR (good)	Cerebellar location, GTR (good)
**5-year OS (%)**	83–87%	>90%

**Table 4:** Characteristics for adult and pediatric craniopharyngioma, based on data from the following references [76,77,78,79,80,81,83,84,85,86,91,98,99,106,107,108] Abbreviations: CP = Craniopharyngioma, OS = overall survival.

Characteristics	Adults	Children
**Age Distribution (years)**	50–70	5–14
**Frequency (%)**	2–5	4–9
**Histology**	Adamantinomatous & Papillary CP	Adamantinomatous CP
**Molecular alterations**	CTNNB1 (aCP), BRAF (pCP)	CTNNB1
**Presenting Symptom**	Visual field deficit	Endocrine disturbances
**Endocrine Deficits at Presentation (%)**	30%	60%
**Primary Treatment**	GTR if possible, radiotherapy, BRAF targeted therapy	Tumor reduction/cyst drainage, proton beam therapy, local chemotherapy(controversial)
**Endocrine Postoperative Complications**	70% diabetes insipidus, 15% growth hormone deficiency	75% growth hormone deficiency, 20% diabetes insipidus
**Visual Field Complications**	7–14% visual field deficits, good postoperative recovery in 60%	8–20% visual field deficits, good postoperative recovery in 50%
**5-year OS (%)**	~90%	~90%

## Data Availability

Not applicable.
